# Functional identification in *Lactobacillus reuteri *of a PocR-like transcription factor regulating glycerol utilization and vitamin B_12 _synthesis

**DOI:** 10.1186/1475-2859-10-55

**Published:** 2011-07-21

**Authors:** Filipe Santos, Jennifer K Spinler, Delphine MA Saulnier, Douwe Molenaar, Bas Teusink, Willem M de Vos, James Versalovic, Jeroen Hugenholtz

**Affiliations:** 1 Center for Integrative Bioinformatics, Vrije Universiteit Amsterdam, Boelelaan 1085, 1081 HV Amsterdam, The Netherlands; 2 TI Food and Nutrition, Kluyver Centre for Genomics of Industrial Fermentation, and NCSB, Nieuwe Kanaal 9A, 6709 PA, Wageningen, The Netherlands; 3 Department of Pathology & Immunology, 1 Baylor Plaza, Baylor College of Medicine, Houston, TX 77030, USA; 4 Department of Pathology, Texas Children's Hospital, 1102 Bates Avenue, Houston, TX 77030, USA; 5 Laboratory of Microbiology, Wageningen University and Research Centre, Dreijenplein 10, 6703 HB Wageningen, The Netherlands; 6 Department of Microbiology, Swammerdam Institute for Life Sciences, University of Amsterdam, Science Park 904, 1098 XH, Amsterdam, The Netherlands; 7 Current Address: NIZO Food Research, Ede, 6710 BA, the Netherlands

## Abstract

**Background:**

*Lactobacillus reuteri *harbors the genes responsible for glycerol utilization and vitamin B_12 _synthesis within a genetic island phylogenetically related to gamma-Proteobacteria. Within this island, resides a gene (*lreu_1750*) that based on its genomic context has been suggested to encode the regulatory protein PocR and presumably control the expression of the neighboring loci. However, this functional assignment is not fully supported by sequence homology, and hitherto, completely lacks experimental confirmation.

**Results:**

In this contribution, we have overexpressed and inactivated the gene encoding the putative PocR in *L. reuteri*. The comparison of these strains provided metabolic and transcriptional evidence that this regulatory protein controls the expression of the operons encoding glycerol utilization and vitamin B_12 _synthesis.

**Conclusions:**

We provide clear experimental evidence for assigning Lreu_1750 as PocR in *Lactobacillus reuteri*. Our genome-wide transcriptional analysis further identifies the loci contained in the PocR regulon. The findings reported here could be used to improve the production-yield of vitamin B_12_, 1,3-propanediol and reuterin, all industrially relevant compounds.

## Background

*Lactobacillus reuteri *is a heterofermentative lactic acid bacterium colonizing the gastrointestinal tract (GI tract) of various mammals, including humans [1]. It is able to convert glycerol to 1,3-propanediol in a two-step enzymatic conversion, yielding NAD^+ ^[2]. In the first reaction, glycerol dehydratase (EC 4.1.2.30), converts glycerol to 3-hydroxypropionaldehyde requiring the presence of vitamin B_12 _as a coenzyme [3]. Reuterin, a mixture of 3-hydroxypropionaldehyde isomers [4], is a potent antimicrobial, bestowing *L. reuteri *with an important growth advantage over other residents of the GI tract, such as Gram-negative enteric bacteria [5,6].

We have shown previously that *L. reuteri *CRL1098 encodes the complete machinery necessary for *de novo *synthesis of vitamin B_12 _in a single chromosomal gene cluster [7]. This cluster was shown to be very similar to that present in various representatives of γ-Proteobacteria, standing out against canonical phylogeny. Complete genome sequence analysis of the type strain of *L. reuteri *revealed that the region immediately upstream of the vitamin B_12 _biosynthesis cluster maintains a gene order similar to that of *Salmonella *[8]. The functionality of this upstream region was demonstrated to also match *Salmonella *where the *pdu *gene cluster is located. The latter encodes the assembly machinery of metabolosomes and the several subunits of a large diol dehydratase that can metabolize both glycerol and 1,2-propanediol [9].

Also within this cluster resides a gene (*lreu_1750*) predicted to encode a 359 amino acid long putative transcription factor of the AraC type family, containing a typical helix-turn-helix domain. Based strictly on its conserved genomic context, this gene has been suggested to encode PocR, a regulatory protein that modulates propanediol utilization (*pdu*) and vitamin B_12 _biosynthesis in enteric bacteria [8-10]. This functional annotation, however, does not seem to be fully supported by sequence homology. And more importantly, to the best of our knowledge, it completely lacks experimental confirmation.

Here we provide the first experimental evidence to support the functional assignment of Lreu_1750. This was achieved by overexpression and inactivation of *lreu_1750*, assessing its impact on central carbon and energy metabolism, and on reuterin and vitamin B_12 _synthesis. In addition, we characterized the genome-wide transcriptional response of both constructs in comparison to their parent strains leading to the identification of the genes comprised in the PocR regulon of *Lactobacillus reuteri*.

## Results and Discussion

### Phylogenetic analysis of Lreu_1750

**Figure 1:**
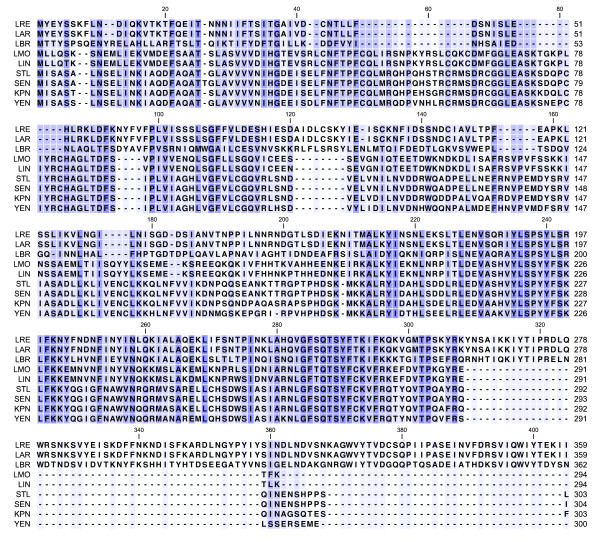
**Amino acid sequence alignment of Lreu_1750 and putatively related sequences**. Alignments were obtained using ClustalW with default settings [20] and visualized in CLC Sequence Viewer 6.5. Abbreviations stand for: LRE, GI:148544956 (*Lactobacillus reuteri *JCM1112); LAR, GI:325683301 (*Lactobacillus reuteri *ATCC PTA 6475); LBR, GI:116334199 (*Lactobacillus brevis *ATCC367); LMO, GI:16410566 (*Listeria monocytogenes *EGDe); LIN, GI:16413573 (*Listeria innocua *Clip11262); STL, GI:16420566 (*Salmonella typhimurium *LT2); SEN, GI:29136962 (*Salmonella enterica *Ty2); KPN, GI:152971720 (*Klebsiella pneumoniae *MGH 78578); YEN, GI:123442942 (*Yersinia enterocolitica *8081). Darker blue background stands for higher percentage conservation of respective residue.

Phylogenetic comparisons between Lreu_1750 and other PocR sequences raise serious doubts about its functional annotation (Figure 1). When compared to the PocR found in enteric bacteria, Lreu_1750 reveals limited amino acid sequence identity (19.1%) and a large percentage of gaps (40.1%). The sequence identity and percentage of gaps (20.5% and 38.8%, respectively) of the PocR-like regulatory proteins of other vitamin B_12_-producing Firmicutes, such as *Listeria monocytogenes*, suggest that it is slightly more related. The closest homolog of Lreu_1750 present in the complete genomes available is found in *L. brevis *ATCC 367 (GI:116334199) with 36.1% identity and only 1.4% gaps. *L. brevis *is also able to produce 1,3-propanediol [3], but it cannot synthesize vitamin B_12 _[7]. The predicted products of *lreu_1750 *and its homolog in *L. brevis *are approximately 60 amino acid residues longer in the C-terminus in comparison to PocR from *S. typhimurium *LT2. This could additionally affect its functionality and further urges the experimental confirmation of its tentative annotation. The putative PocR of two different wild type *L. reuteri *strains, JCM1112 and ATCC PTA 6475, have also been aligned and found to display 100% sequence identity and 0% gaps (Figure 1). Subsequent experiments have been carried out using derivatives of both strains predominantly for technical convenience. Nevertheless, this choice is also important to further substantiate the generality of our findings regarding the role of this PocR-like protein in *L. reuteri *strains.

### Physiological effects

**Figure 2:**
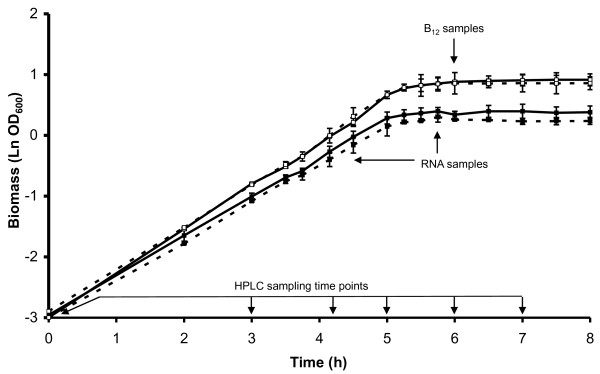
**Biomass formation and sampling scheme in pH-controlled batch fermentations**. Experiments were carried out in CDM in the presence (white squares) or absence (black square) of glycerol by *L. reuteri *transformed with pNZ7021 (empty plasmid, solid lines) or pNZ7748 (harboring *lreu_1750*, dashed lines).

**Figure 3:**
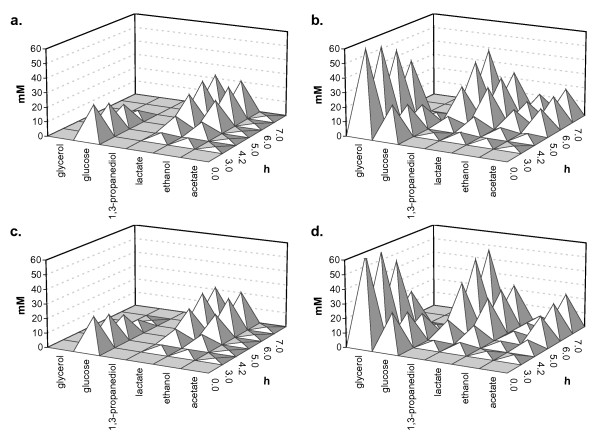
**Substrate consumption and product formation in pH-controlled batch fermentations**. Substrate consumption and product formation by different constructs of *L. reuteri *in CDM and in CDM with 0.5% glycerol (v/v). a. *L. reuteri *pNZ7021 (empty plasmid) in CDM; b. *L. reuteri *pNZ7021 in CDM with glycerol; c. *L. reuteri *pNZ7748 (harboring *lreu_1750*) in CDM; d. *L. reuteri *pNZ7748 in CDM with glycerol.

The functional assignment of *lreu_1750 *was initiated by characterizing the impacts of its overexpression on central carbon and energy metabolism. Since functionally active glycerol metabolizing enzymes are encoded in the vicinity of *lreu_1750*, the experimental focus was on glycerol metabolism. In the absence of glycerol, except for a slight impairment (< 10%) of *μ*_max _(Figure 2), no metabolic effects were observed related to the overexpression of *lreu_1750 *(Figure 3, panels a. and c.). In the presence of glycerol, however, the overexpressing strain in comparison with JCM1112 transformed with pNZ7021 (empty plasmid), displays a drop in the final ethanol concentration from 13.7 to 6.2 mM while acetate increased approximately 4 mM. This enhancement of the shift from ethanol to acetate formation (*p*-value < 0.025, paired two-tailed *t*-test) is accompanied by a 22.5% increase of 1,3-propanediol production, which is produced on a 2:1 molar ratio with acetate, assuring the regeneration of reducing equivalents (Figure 3, panels b. and d.).

**Figure 4:**
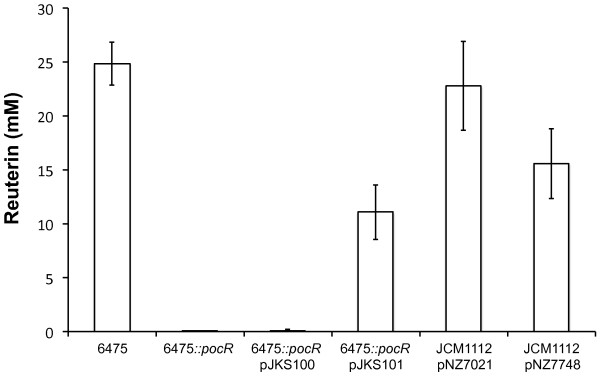
**Reuterin production by *L. reuteri *strains in MRS media**. Bars represent average values along with standard deviation (error bars) of at least three independent biological replicates using strains 6475 (parent strain), 6475*::pocR *(PocR deficient mutant), 6475*::pocR *pJKS100 (PocR deficient mutant transformed with empty plasmid as negative control), 6475*::pocR *pJKS101 (PocR deficient mutant complemented with putative *pocR *of 6475 under control of its native promoter), JCM1112 pNZ7021 (type strain transformed with empty plasmid) and JCM1112 pNZ7748 (type strain carrying the *lreu_1750 *overexpression). Experiment was performed at least twice with similar results.

Overexpressing Lreu_1750 does not lead to significant changes (*p*-value = 0.07, paired two-tailed *t*-test) in reuterin production (Figure 4). This is not totally unexpected, since changes in the level of enzymes involved in central carbon metabolism often do not result in drastic changes in fluxes [11]. In contrast, the disruption of the *lreu_1750 *gene leads to an abrupt decrease in reuterin production from 25 mM in the parent strain to undetectable levels (< 0.1 mM) in the mutant. When this strain is complemented with a plasmid harboring *lreu_1750 *under control of its native promoter (pJKS101), reuterin production is restored to levels in the same order of magnitude as *L. reuteri *6475 (11 mM). Mostly human-derived *L. reuteri *strains can produce reuterin, and therefore, it is thought that this may be important for their survival in the human GI tract [1]. The observed reduction by more than 250-fold in reuterin production most likely debilitates the probiotic functionality of the PocR mutant strain [1,12]. Furthermore, it will condition the potential utilization of glycerol for the regeneration of NAD+, limiting its biomass yield on carbon substrate [10].

**Figure 5:**
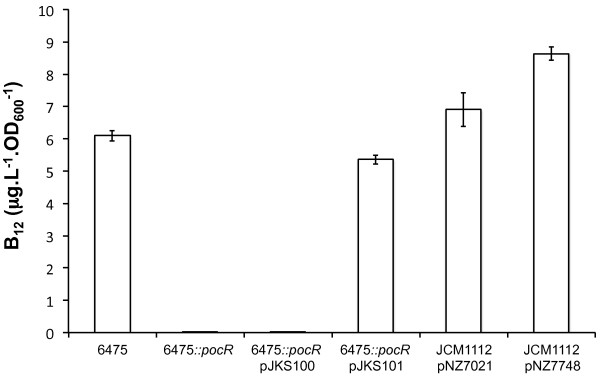
**Vitamin B_12 _production by *L. reuteri *strains in commercial Vitamin B_12_-assay medium supplemented with glycerol**. Bars represent average values along with standard deviation (error bars) of at least two independent biological replicates measured in triplicate using strains 6475 (parent strain), 6475*::pocR *(PocR deficient mutant), 6475*::pocR *pJKS100 (PocR deficient mutant transformed with empty plasmid), 6475*::pocR *pJKS101 (PocR deficient mutant complemented with putative *pocR *of 6475 under control of its native promoter), JCM1112 pNZ7021 (type strain transformed with empty plasmid) and JCM1112 pNZ7748 (type strain carrying the *lreu_1750 *overexpression).

The regulatory role of Lreu_1750 on vitamin B_12 _synthesis is clearly illustrated by the drastic inhibitory effect that its inactivation exerts over vitamin B_12 _production (Figure 5). In contrast to the parent strain, the deletion mutant of the putative PocR did not produce detectable levels of B_12 _(6.09 and less than 0.01 μg.L^-1^.OD_600_^-1^, respectively). Furthermore, the complementation of the mutant with pJKS101 (harboring the putative *pocR*) leads to the reestablishment of B_12 _production (5.36 μg.L^-1^.OD_600_^-1^). Additionally, in the strain overexpressing Lreu_1750 (JCM1112 pNZ7748) we observe a significant increase (*p*-value < 0.016, paired two-tailed *t*-test) of more than 25% in vitamin B_12 _production in comparison to JCM1112 transformed with the empty plasmid (pNZ7021). This increase was obtained regardless of the addition of glycerol, and was observed in all the media and conditions tested including the pH-controlled batch fermentations using CDM (data not shown).

The physiological effects observed for the overexpression and inactivation of Lreu_1750 are all in agreement with its functional assignment as the regulatory protein PocR.

### Transcriptomic response

In order to probe the global regulatory role of the putative PocR of *L. reuteri*, we compared the transcriptomes of the deficient and overexpressing strains relative to their parent strains. Considering that *(i) *glycerol has been shown to induce the expression of *lreu_1750 *[10], masking the effect of its overexpression; *(ii) *consequently the differentiating phenotype of the PocR deficient strain can be best observed under conditions in which its growth kinetics are hampered (such as in the presence of glycerol - Figure 2); and *(iii) *there is a large redundancy between the different transcriptome analyses carried out; most emphasis in this report has been put on the data related to the *lreu_1750 *overexpression in the absence of glycerol. The complete list of differentially regulated genes under all conditions assayed is available in Additional file 1: Transcriptome analysis data.

**Figure 6:**
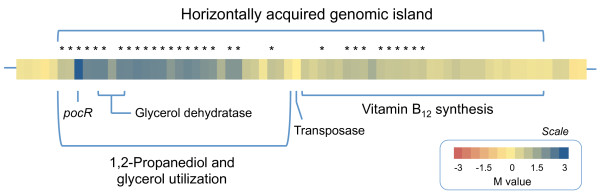
**Relative expression levels of the loci situated within the genetic island that harbors PocR**. Color-scale plot of the relative expression levels of the loci situated between *lreu_1690 *and *lreu_1757 *(not to scale). All genes situated within the genetic island are up-regulated when *lreu_1750 *is overexpressed with the exception of the transposase. The lack of statistical significance observed for some of the loci is discussed in the text. Abbreviations stand for: M, log_2_(intensity of signal of *L. reuteri *pNZ7748/intensity of signal of *L. reuteri *pNZ7021); "*", *p*-value ≤ 0.05.

Upon overexpressing *lreu_1750 *only 120 genes (approximately 6% of the genome) are differentially regulated, of which, all but two are up-regulated. Three functional classes were represented with 10% or more of its members in the list of differentially expressed loci, namely the ones related to coenzymes, secondary metabolites and energy production (Additional file 1, Table S2). A closer inspection of the list of differentially regulated genes shows that *lreu_1750 *is clearly involved in the regulation of the same processes that have been linked to PocR in *Salmonella *[13]. These genes include the *pdu *cluster flanking *lreu_1750*, encoding the several subunits of the diol dehydratase and the metabolosome-assembly proteins [9], along with the two operons of the B_12 _biosynthesis cluster [7] (Figure 6). The lack of statistical significance observed for some of the expression data of the B_12 _synthesis cluster is easily explained. This cluster is divided into two multicistronic operons with a relatively low abundance and a remarkably large size. This raises great technical difficulties during mRNA purification as reported in the past [7,14].

Besides these genes stretching from *lreu_1695 *to *lreu_1752*, which are expected to be regulated by PocR by homology with *Salmonella *[2], we found two genes (*lreu_0429 *and *lreu_0430*) predicted to be co-transcribed and annotated with unknown function in GenBank, that are up-regulated ~3.5-fold (Table 1). A closer look at their sequence indicates that these are most likely two subunits of an ATPase transporter [15], which have been tentatively associated with copper transport in ERGO [16]. We speculate that these are cobalt- rather than copper-transporters, based on the fact that cobalt availability is essential for the synthesis of vitamin B_12_. We also found regulated a few genes related to sugar uptake and carbon metabolism. These are presumably related to the up-regulation of *lreu_0088 *(transcription factor of LacI family), which is most likely a consequence of a slight drop in growth rate caused by the extra burden of the *lreu_1750 *overexpression (Figure 2). A considerable number of enzymes involved in recombination and DNA repair were also up-regulated. This is probably a consequence of the homologous region to the genome of *L. reuteri *present in plasmid pNZ7748 (harboring *lreu_1750*) and not in pNZ7021 (empty vector), as previously observed [17,18].

**Table 1:** Relative expression levels of loci associated to PocR and not within its flanking region^***a.***^

Locus	Function	**M**^***b.***^	*p^c.^*	Accession number
Lreu_0088	Transcriptional regulator, LacI family	1.11	0.03	gi|148543330
Lreu_0103	3-hydroxybutyryl-CoA dehydrogenase (EC 1.1.1.157)	0.89	0.03	gi|148543342
Lreu_0429	Putative cobalt-transporting ATPase^*d.*^	1.85	0.00	gi|148543665
Lreu_0430	Putative cobalt-transporting ATPase^*d.*^	1.76	0.00	gi|148543666
Lreu_0479	Arabinose-proton symporter	1.67	0.00	gi|148543714
Lreu_0631	Pyruvate dehydrogenase alpha subunit (EC 1.2.4.1)	0.88	0.02	gi|148543863
Lreu_0632	Pyruvate dehydrogenase beta subunit (EC 1.2.4.1)	0.86	0.04	gi|148543864
Lreu_0633	Dihydrolipoamide acetyltransferase component of pyruvate dehydrogenase complex (EC 2.3.1.12)	0.81	0.03	gi|148543865
Lreu_0910	Alpha-galactosidase (EC 3.2.1.22)	0.90	0.03	gi|148544139
Lreu_1007	Transcription regulator, Crp family	-0.82	0.04	gi|148544234
Lreu_1531	Fumarate hydratase (EC 4.2.1.2)	1.09	0.05	gi|148544743
Lreu_1768	Lactose permease	0.97	0.05	gi|148544974
Lreu_1832	Histidine decarboxylase (EC 4.1.1.22)	-1.67	0.03	gi|148545038

*^a. ^*Genes predicted to encode phage-related proteins, recombinases, mobile elements, DNA repair and general or unknown functions were omitted (for full list, please see Additional file 1, Table S1)

*^b. ^*M, log_2_(intensity of signal of *L. reuteri *pNZ7748/intensity of signal of *L. reuteri *pNZ7021).

*^c. ^p, p*-value.

*^d. ^*Annotated as hypothetical protein in GenBank and as copper-transporting ATPase (EC 3.6.3.10) in ERGO database [16].

The transcriptome studies carried out for the PocR insertion mutant were consistent with the results obtained through the overexpression of *lreu_1750*. We mainly observed in the PocR mutant compared to the wild-type strain, a down-regulation of the genes located within the genetic island that comprises the *pdu *and vitamin B_12 _operons (Additional file 1, Table S3). Again due to the rarity and fragility of these transcripts [7,14] only 30 out of 58 loci are differentially expressed significantly (*p*-value ≤ 0.05) even though the whole region, excluding the transposase, appears co-regulated.

There is strong phylogenetic evidence supporting that the *pdu *and vitamin B_12 _synthesis gene clusters have been acquired by *L. reuteri *through distant horizontal gene transfer [7,8]. The confinement of the PocR regulon to mostly one continuous stretch of the chromosome (Figure 6), with exception of the putative cobalt transporter, further substantiates this hypothesis.

## Conclusions

In this study, we have provided experimental evidence that *lreu_1750 *encodes a PocR-like regulatory protein, despite its lack of sequence homology to PocR from enteric bacteria. This was achieved by overexpression and inactivation of *lreu_1750*, and assessment of its impact on central carbon and energy metabolism, and on reuterin, 1,3-propanediol and vitamin B_12 _biosynthesis. In addition, we characterized the genome-wide transcriptional response of both constructs in comparison to the wild-type leading to the identification of the genes encompassed in the PocR-like regulon of *L. reuteri*. The latter were found to be similar to the ones present in some representatives of γ-Proteobacteria. Ultimately, the demonstrated stimulatory effects of PocR on vitamin B_12_, 1,3-propanediol and reuterin synthesis could be applied to improving the production yield of these industrially relevant compounds.

## Methods

### Phylogenetic analysis of Lreu_1750

The sequence of Lreu_1750 (GI:148544956) was entered as a string to search for closely related homologs within available microbial genomes using the protein-protein BLAST algorithm [19]. Relevant sequences were retrieved and aligned using ClustalW with default settings [20] and visualized in CLC Sequence Viewer 6.5.

### Strains, plasmids, primers and cultivation conditions

The bacterial strains, plasmids and primers used in this study are listed in Table 2. *L. reuteri *strains were cultivated at 37°C in undefined MRS broth [21], in Vitamin B_12 _assay medium (Sigma-Aldrich, Zwijndrecht, The Netherlands) enriched with 0.5% glycerol (v/v), in the semi-defined medium LDMIIIG [12] and in a chemically defined medium (CDM) previously used to study vitamin B_12 _production in *L. reuteri *[14]. When appropriate, erythromycin and/or chloroamphenicol were added to a final concentration of 10 μg/mL.

**Table 2:** Strains, plasmids and primers used in this study

Materials	Relevant features	Source or reference
***Strains***		
*L. reuteri* JCM1112	Type strain, synonymous to ATCC 23272, DSM 20016 and F275. Human isolate.	Japanese Collection of Microorganisms (Riken, Japan)
*L. reuteri* ATCC PTA 6475	Synonymous to MM4-1A. Finnish mother's milk isolate.	Biogaia AB (Stockholm, Sweden)
6475*::pocR*	Em^R^, *pocR *insertion mutant derivative of *L. reuteri *ATCC PTA 6475	This study
*Lc. lactis* NZ9000	MG1363 *pepN:nisRK*, cloning host.	NIZO culture collection (Ede, The Netherlands)
*L. delbrueckii* NIZO235	*L. delbrueckii *subsp. *lactis *ATCC 7830. Vitamin B_12 _assay indicator strain.	NIZO culture collection (Ede, The Netherlands)

***Plasmids***		
pCR^®^2.1	Used in routine cloning and to construct pJKS100	Invitrogen (Carlsbad, CA)
pLEM5	*L. reuteri *replication origin used to construct pJKS100	[28]
pNZ7021	Cm^R^, pNZ8148 derivative with the nisin promoter replaced by the *pepN *promoter	[23]
pNZ7748	Cm^R^, pNZ7021 derivative harboring *lreu_1750 *downstream of the *pepN *promoter.	This study
pVE6007	Cm^R^, repA-positive temperature-sensitive derivative of pWV01	[27]
pORI28	Em^R^, repA-negative derivative of pWV01	[35]
pORIpocR	Em^R^, pORI28 derivative harboring internal fragment of gene encoding putative PocR	This study
pJKS100	Cm^R^, *E. coli-L. reuteri *shuttle vector	This study
pJKS101	Cm^R^, pJKS100 derivative expressing 6475 *pocR *gene under control of its natural promoter	This study

***Primers***	***5' - 3'***	***Application***
P180	AAAAGGTACCGTAGGCGAAATTCAAATGTACG	Amplification of *lreu_1750 *and addition of *Kpn*I site
P181	GAATAAATAAGAGGCTGGGCAC	Amplification of *lreu_1750*
P182	ATGAACTCTATTCAGGAATTG	Control of pNZ7748
LR0062F-BHI	TGACGGATCC**TAA**CACAAGCATTACCGGAGCAATTG	Amplification of internal fragment of putative *pocR*, addition of *Bam*HI site and translational stop codon
LR0062R-ERI	TGACGAATTCGCGTCTGATTCTATATGTGATTC	Amplification of internal fragment of putative *pocR *and addition of *Eco*RI site
LR0062 FL F	CGCTTTATCCTCAATTTGTTACG	Amplification of wild-type *pocR *gene and natural promoter
LR0062 FL R	GCTTTTACCATTGCATCAGCAG	Amplification of wild-type *pocR *gene and natural promoter

### Construction of putative *pocR *overexpression and deletion mutants

Gene *lreu_1750*, encoding the putative PocR in JCM1112^T^, was overexpressed constitutively under control of the *pepN *promoter in a similar fashion as previously described [22]. A fragment containing *lreu_1750 *was amplified from chromosomal DNA of *L. reuteri *using Herculase II DNA polymerase (Stratagene, La Jolla, USA), and primers P180 and P181 (Table 2). After digestion with *Kpn*I, the modified amplicon was purified and cloned in pNZ7021 making use of the *Kpn*I and *Pml*I restriction sites directly downstream of the *pepN *promoter. The resulting plasmid, termed pNZ7748, was used directly from the ligation reactions to transform *Lactococcus lactis *NZ9000 by electroporation [23]. Subsequently, pNZ7748 was purified from *Lc. lactis *as previously described [24] and, after confirming the sequence of the insert using both P181 and P182, it was used to transform *L. reuteri *also by electroporation [25].

The disruption of the putative *pocR *gene was carried out in *L. reuteri *ATCC PTA 6475, which shares an identical sequence with the type strain (JCM1112) for this region of the chromosome [1]. This was achieved by site-specific integration of plasmid pORIpocR as described previously [26] using the temperature-sensitive plasmid pVE6007 [27] as the helper plasmid. The internal fragment of the target gene was amplified by PCR using primers LR0062F-BHI and LR0062R-ERI (Table 2), and inserted into pORI28 by directional cloning using standard techniques [24]. The resulting insertion mutant was designated 6475::*pocR*.

### Complementation of *L. reuteri *6475*::pocR*

An *E. coli*-*L. reuteri *shuttle vector (pJKS100) was constructed by combining an *L. reuteri *replicon from pLEM5 [28], the chloramphenicol resistance gene (CmR) from pVE6007 [27], the *L. lactis *promoter (P_23_) [29], and the pUC origin and multiple cloning site (MCS) from pCR^®^2.1 (Invitrogen, Carlsbad, CA). Each fragment was PCR amplified from their respective template, restriction enzyme digested and subsequently ligated to generate the final shuttle-vector, pJKS100. To create the complementation vector for 6475::*pocR*, the *L. reuteri *6475 *pocR *gene with its natural promoter was PCR-amplified from genomic DNA using LR0062 FL F and LR0062 FL R primers and cloned into pJKS100 using standard techniques [24]. Both constructs, pJKS100 and pJKS101, were electroporated seperately into *L. reuteri *6475::*pocR *as previously described [25].

### Fermentation conditions and substrate and product analysis

The physiological effects of the overexpression of *lreu_1750 *were studied in pH-controlled batch cultivations of *L. reuteri *pNZ7748 (*lreu_1750 *overexpression) and *L. reuteri *pNZ7021 (empty plasmid) in CDM in the presence or absence of glycerol carried out as described previously [14]. At different time points, samples were taken for transcriptome, supernatant and vitamin B_12 _analysis (Figure 2). We determined the extracellular concentration of main fermentation substrates and products by HPLC, as described elsewhere [6,30].

The comparison between the insertion mutant, 6475*::pocR*, and its parent strain was established in batch fermentations of LDMIIIG or MRS carried out in an anaerobic chamber (80% N_2_, 10% H_2_, and 10% CO_2_; Microbiology International). Transcriptome comparisons were carried out at the end of the fermentation when biomass concentration became stable.

### Vitamin B_12 _and reuterin determination

Vitamin B_12 _levels were determined as described in the Official methods of analysis of AOAC International [31], using a bioassay with *L. delbrueckii *subsp. *lactis *ATCC 7830 as the indicator strain. Reuterin production was measured with a bioassay and carried out as previously described [5].

### Transcriptome analyses

#### - Transcriptional analysis of the putative PocR overexpression mutant

The transcriptome of cells transformed with pNZ7748 (*lreu_1750 *overexpression) and pNZ7021 (empty plasmid) were compared using cDNA microarrays as previously detailed [14] using a hybridization scheme comprising 17 arrays in a loop-design. The following samples were hybridized per array labeled with cyanine3 and cyanine5, respectively: sta-F6 and sta-F5, sta-F7 and sta-F8, sta-F5 and sta-F7, sta-F8 and sta-F6, sta-F3 and sta-F4, sta-F1 and sta-F3, sta-F2 and sta-F1, sta-F4 and sta-F2, exp-F3 and exp-F4, exp-F1 and exp-F3, exp-F2 and exp-F1, exp-F4 and exp-F2, exp-F4 and sta-F4, sta-F3 and exp-F3, sta-F2 and sta-F8, sta-F4 and sta-F6, exp-F2 and sta-F2. Here, F1 and F5 represent completely independent biological duplicates of *L. reuteri *pNZ7021 cultured in the absence of glycerol; F2 and F6 represent completely independent biological duplicates of *L. reuteri *pNZ7748 cultured in the absence of glycerol; F3 and F7 represent completely independent biological duplicates of *L. reuteri *pNZ7021 cultured in the presence of glycerol; and F4 and F8 represent completely independent biological duplicates of *L. reuteri *pNZ7748 cultured in the presence of glycerol. The prefix exp- and sta- stand for cells harvested at mid-logarithmic and early-stationary growth phases, respectively. The custom probe design of the Agilent 11 K microarray platform (Agilent Technologies, Santa Clara, CA, USA) used is available at the Gene Expression Omnibus http://www.ncbi.nlm.nih.gov/geo under accession number GPL6856, and the data obtained were deposited in the same repository under accession number GSE13289.

#### - Transcriptional analysis of the putative PocR insertion mutant

The transcriptome of the insertion mutant, 6475*::pocR*, and its parent strain were compared using two-color microarrays as previously detailed [32]. Briefly, oligonucleotides (60-mers) were designed and synthesized for 1,966 open reading frames from a draft genome sequence of *L. reuteri *ATCC PTA 6475 [1]. For expression analyses, three biological replicates of the insertion mutant and parent strain were compared. Moreover, dye-swap hybridization was performed for each comparison. Following mRNA isolation [32], cDNA synthesis, labeling, and hybridization were performed as previously described [32]. Information regarding the microarray platform and data obtained is deposited at NCBI Gene Expression Omnibus (GEO; http://www.ncbi.nlm.nih.gov/geo) under GPL7541 and GSE22926, respectively.

GenePix Pro 4.0.12 software was utilized for image analysis of the 6475 microarrays. Normalization within arrays and between arrays was performed by applying the Loess algorithm [33] using the Limma package [34] in R http://www.r-project.org. Normalized intensities were used for further analysis. The average signal intensities of three biological replicates were calculated in order to compare the relative gene expression of mutant and wild type strains. The statistical significance of differences was calculated based on variation in biological duplicates, using the eBayes function in Limma (cross-probe variance estimation) and false discovery rate (FDR) adjustment of the *p*-values. Only genes that were differentially expressed by least 1.5-fold with FDR-adjusted *p*-values lower than 0.05 were considered significant.

## Competing interests

The authors declare that they have no competing interests.

## Authors' contributions

FS performed the phylogenetic analysis and all experiments related to the overexpression of PocR and measurement of vitamin B_12 _production, and wrote the first draft of the manuscript. JS constructed the PocR mutant and measured reuterin production and helped to draft the manuscript. DS performed the fermentations and cDNA microarrays related to the PocR mutant and helped to draft the manuscript. DM supervised the statistical analysis and interpretation of the cDNA microarrays related to the PocR overexpression. BT supervised the interpretation of the fermentation data. WV helped in the design and supervision of the PocR overexpression experiments and helped to draft the manuscript. JV helped in the design and supervision of the PocR deletion experiments and helped to draft the manuscript. JH conceived the study, participated in its coordination and helped to draft the manuscript. All authors read and approved the final manuscript.

## Supplementary Material

Additional file 1 **Transcriptome analysis data**. Single PDF containing three tables with additional transcriptome analysis data: Additional file 1, Table S1, Complete list of transcripts from *L. reuteri *that are differentially expressed by the overexpression of *lreu_1750*; Additional file 1, Table S2, Distribution of transcripts listed in Additional file 1, Table S1 throughout categories of clusters of orthologuous groups (COG, [35]); Additional file 1, Table S3, Complete list of loci from *L. reuteri *ATCC PTA 6475 that are differentially expressed by the disruption of the putative PocR. Click here for file
