# Supplementary material for: Functional identification in Lactobacillus reuteri of a PocR-like transcription factor regulating glycerol utilization and vitamin B12 synthesis
Source: Microb Cell Fact. 2011 Jul 21;10:55. doi: 10.1186/1475-2859-10-55 (PMC3162504; doi:10.1186/1475-2859-10-55)
Supplement: Additional file 1 — Transcriptome analysis data. Single PDF containing three tables with additional transcriptome analysis data: Additional file 1, Table S1, Complete list of transcripts from L. reuteri that are differentially expressed by the overexpression of lreu_1750; Additional file 1, Table S2, Distribution of transcripts listed in Additional file 1, Table S1 throughout categories of clusters of orthologuous groups (COG, [35]); Additional file 1, Table S3, Complete list of loci from L. reuteri ATCC PTA 6475 that are differentially expressed by the disruption of the putative PocR. [file 1475-2859-10-55-S1.PDF]

Functional identification in *Lactobacillus reuteri* of a PocR-like transcription factor regulating glycerol utilization and vitamin B_{12} synthesis

Filipe Santos, Jennifer K. Spinler, Delphine M. A. Saulnier, *et al.*

Additional File 1 - Transcriptome analysis data

Content

Table S1. Complete list of transcripts from *L. reuteri* JCM1112^T that are differentially expressed by the overexpression of *lreu_1750*. Comparisons were established for cells cultured in CDM and in CDM supplemented with glycerol. Page 2.

Table S2. Distribution of transcripts listed in Table S1 throughout categories of clusters of orthologous groups (COG). Page 5.

Table S3. Complete list of loci from *L. reuteri* ATCC PTA 6475 that are differentially expressed by the disruption of the putative PocR. Page 6.

Table S1. Complete list of transcripts from *L. reuteri* JCM1112[T] that are differentially expressed by the overexpression of *lreu_1750*[a]. Comparisons were established for cells cultured in CDM and in CDM supplemented with glycerol.

Locus	Function[b]	CDM		CDMglycerol		COG	Accession number
		M[c]	p[d]	M[c]	p[d]		
Lreu_0539	Thioredoxin			0.97	0.02	C	gi|148543774
Lreu_0631	Pyruvate dehydrogenase E1 component alpha subunit (EC 1.2.4.1)	0.88	0.02	-1.53	0.00	C	gi|148543863
Lreu_0632	Pyruvate dehydrogenase E1 component beta subunit (EC 1.2.4.1)	0.86	0.04	-1.66	0.00	C	gi|148543864
Lreu_0633	Dihydrolipoamide acetyltransferase component of pyruvate dehydrogenase complex (EC 2.3.1.12)	0.81	0.03	-1.44	0.00	C	gi|148543865
Lreu_0634	Dihydrolipoamide dehydrogenase (EC 1.8.1.4)			-1.17	0.02	C	gi|148543866
Lreu_1531	Fumarate hydratase (EC 4.2.1.2)	1.09	0.05			C	gi|148544743
Lreu_1727	Flavodoxin	0.92	0.01			C	gi|148544933
Lreu_1734	Propanol dehydrogenase (EC 1.1.1.-)	1.86	0.00			C	gi|148544940
Lreu_1735	Propionaldehyde dehydrogenase [CoA-acylating] (EC 1.2.1.-)	1.94	0.00			C	gi|148544941
Lreu_0098	Glutamine-binding protein / Glutamine transport system permease protein glnP			1.23	0.00	E	gi|148543337
Lreu_1544	Xaa-His dipeptidase (EC 3.4.13.3)			-0.94	0.01	E	gi|148544756
Lreu_1728	Propanediol utilization protein pduV			-1.05	0.02	E	gi|148544934
Lreu_1732	Ethanolamine utilization protein eutS	1.87	0.00			E	gi|148544938
Lreu_1748	Propanediol utilization protein pduB	2.22	0.00			E	gi|148544954
Lreu_1751	Ethanolamine utilization protein eutJ	0.87	0.03			E	gi|148544957
Lreu_0111	Deoxyribose-phosphate aldolase (EC 4.1.2.4)			-1.90	0.00	F	gi|148543350
Lreu_0114	Purine nucleoside phosphorylase II (EC 2.4.2.1)			-1.75	0.00	F	gi|148543353
Lreu_0112	Phosphopentomutase (EC 5.4.2.7)			-1.89	0.00	G	gi|148543351
Lreu_0288	Beta-galactosidase large subunit (EC 3.2.1.23)			-1.27	0.00	G	gi|148543525
Lreu_0289	Beta-galactosidase small subunit (EC 3.2.1.23)			-1.21	0.00	G	gi|148543526
Lreu_0418	Glucose uptake protein homolog			-0.87	0.03	G	gi|148543654
Lreu_0910	Alpha-galactosidase (EC 3.2.1.22)	0.90	0.03			G	gi|148544139
Lreu_1017	Alpha-glucosidase (EC 3.2.1.20)			-1.64	0.00	G	gi|148544244
Lreu_1538	Aldose 1-epimerase (EC 5.1.3.3)			-0.89	0.02	G	gi|148544750
Lreu_1752	Glycerol uptake facilitator protein	0.93	0.02			G	gi|148544958
Lreu_1768	Lactose permease	0.97	0.05	-1.49	0.00	G	gi|148544974
Lreu_1776	Galactose-1-phosphate uridylyltransferase (EC 2.7.7.10)			-0.97	0.04	G	gi|148544982
Lreu_0155	Nicotinate phosphoribosyltransferase (EC 2.4.2.11)			0.98	0.04	H	gi|148543394
Lreu_1677	Lipoate-protein ligase A (EC 6.3.2.-)			-1.17	0.01	H	gi|148544883
Lreu_1705	Adenosylcobyric acid synthase (glutamine-hydrolysing) (EC 6.3.5.10)			0.96	0.03	H	gi|148544911
Lreu_1710	Precorrin-2 C20-methyltransferase (EC 2.1.1.130)			0.96	0.01	H	gi|148544916
Lreu_1711	Sirohydrochlorin cobaltochelatase (EC 4.99.1.3)	0.83	0.05	1.03	0.01	H	gi|148544917
Lreu_1712	Uroporphyrin-III C-methyltransferase (EC 2.1.1.107) / Uroporphyrinogen-III synthase (EC 4.2.1.75)	0.86	0.03	0.82	0.05	H	gi|148544918
Lreu_1713	Precorrin-6X reductase (EC 1.3.1.54)	0.84	0.04			H	gi|148544919
Lreu_1714	Precorrin-3B C17-methyltransferase (EC 2.1.1.131)	0.93	0.05			H	gi|148544920
Lreu_1716	Precorrin-4 C11-methyltransferase (EC 2.1.1.133)	0.98	0.02			H	gi|148544922
Lreu_1717	Precorrin-6Y C5,15-methyltransferase [decarboxylating] subunit CbiT (EC 2.1.1.132)	1.04	0.02			H	gi|148544923
Lreu_1718	Precorrin-6Y C5,15-methyltransferase [decarboxylating] subunit CbiE (EC 2.1.1.132)	1.10	0.02			H	gi|148544924
Lreu_0103	3-hydroxybutyryl-CoA dehydrogenase (EC 1.1.1.157)	0.89	0.03			I	gi|148543342
Lreu_0088	Transcriptional regulator, LacI family	1.11	0.03			K	gi|148543330
Lreu_0801	Transcriptional regulator, Cro/CI family			0.81	0.04	K	gi|148544031
Lreu_0831		1.21	0.01	1.77	0.00	K	gi|148544061
Lreu_1109	Phage tail protein	2.29	0.00	1.48	0.00	K	gi|148544333
Lreu_1111	Phage protein	2.00	0.00	1.44	0.01	K	gi|148544335
Lreu_1112		2.18	0.00	1.69	0.00	K	gi|148544336
Lreu_1750	Transcriptional regulator, AraC family	3.11	0.00	3.58	0.00	K	gi|148544956
Lreu_1796	Transcriptional regulator			0.84	0.05	K	gi|148545002
Lreu_0383	Excinuclease ABC subunit B	1.05	0.05			L	gi|148543619
Lreu_0384	Excinuclease ABC subunit A	0.94	0.03			L	gi|148543620
Lreu_0523	RecA protein			1.18	0.02	L	gi|148543758
Lreu_0796	Site-specific recombinase	1.32	0.04			L	gi|148544026
Lreu_0811	RecT protein	1.95	0.00	1.73	0.00	L	gi|148544041
Lreu_1120	Terminase small subunit	2.28	0.00	1.46	0.00	L	gi|148544344
Lreu_1139	RecT protein	1.76	0.02	1.55	0.03	L	gi|148544363
Lreu_1114	Phage protein	2.15	0.00	1.61	0.00	M	gi|148544338

Locus	Function[b]	CDM		CDMglycerol		COG	Accession number
		M[c]	p[d]	M[c]	p[d]		
Lreu_0539	Thioredoxin			0.97	0.02	O	gi|148543774
Lreu_0891	ATP-dependent transporter sufC			-0.81	0.03	O	gi|148544121
Lreu_0216	Manganese-binding protein			-1.68	0.05	P	gi|148543453
Lreu_0217	Manganese transport system ATP-binding protein			-1.44	0.02	P	gi|148543454
Lreu_1650	Non-specific DNA-binding protein Dps / Iron-binding ferritin-like antioxidant protein / Ferroxidase (EC 1.16.3.1)	1.08	0.02	0.83	0.04	P	gi|148544857
Lreu_1706	Cobalt transport ATP-binding protein cbiO			1.06	0.03	P	gi|148544912
Lreu_1707	Cobalt transport protein cbiQ			1.14	0.03	P	gi|148544913
Lreu_1708	Cobalt transport protein cbiN			1.14	0.03	P	gi|148544914
Lreu_1709	CbiM protein	0.81	0.04	1.04	0.01	P	gi|148544915
Lreu_0156	Pyrazinamidase (EC 3.5.1.-) / Nicotinamidase (EC 3.5.1.19)			1.04	0.04	Q	gi|148543395
Lreu_1738	Ethanolamine utilization protein eutN	2.05	0.00			Q	gi|148544944
Lreu_1740	PduL	2.17	0.00			Q	gi|148544946
Lreu_1741	Propanediol utilization protein pduA	2.18	0.00			Q	gi|148544947
Lreu_1742		2.23	0.00			Q	gi|148544948
Lreu_1743		2.29	0.00			Q	gi|148544949
Lreu_1744	Glycerol dehydratase reactivation factor large subunit	2.18	0.00			Q	gi|148544950
Lreu_1745	Diol dehydratase gamma subunit (EC 4.2.1.28)	2.20	0.00			Q	gi|148544951
Lreu_1747	Diol dehydratase large subunit (EC 4.2.1.28)	2.23	0.00			Q	gi|148544953
Lreu_1749	Propanediol utilization protein pduA	2.18	0.00			Q	gi|148544955
Lreu_0419	O-acetyltransferase (EC 2.3.1.-)			-0.93	0.04	R	gi|148543655
Lreu_0479	Arabinose-proton symporter	1.67	0.00			R	gi|148543714
Lreu_0833	Terminase large subunit	1.30	0.01	1.67	0.00	R	gi|148544063
Lreu_1117	Terminase large subunit	2.13	0.00	1.37	0.01	R	gi|148544341
Lreu_1795	ATPase associated with chromosome architecture/replication	1.35	0.01			R	gi|148545001
Lreu_0429	Copper-transporting ATPase (EC 3.6.3.10)	1.85	0.00	0.87	0.03	S	gi|148543665
Lreu_0430	Copper-transporting ATPase (EC 3.6.3.10)	1.76	0.00	0.86	0.04	S	gi|148543666
Lreu_0803	Phage antirepressor protein	1.60	0.00	1.70	0.00	S	gi|148544033
Lreu_0838	Major capsid protein	1.16	0.04	1.65	0.01	S	gi|148544068
Lreu_0846	Phage protein	1.47	0.00	1.50	0.00	S	gi|148544076
Lreu_1104	Phage protein	1.92	0.00	1.30	0.02	S	gi|148544328
Lreu_1106	Phage protein	2.50	0.00	1.32	0.02	S	gi|148544330
Lreu_1110	Phage tail protein	2.41	0.00	1.47	0.00	S	gi|148544334
Lreu_1116	Portal protein	2.17	0.00	1.32	0.01	S	gi|148544340
Lreu_1142	Phage antirepressor protein	1.62	0.00	1.70	0.00	S	gi|148544366
Lreu_1545	Arginine/ornithine antiporter			-1.57	0.00	S	gi|148544757
Lreu_1629	Integral membrane protein	1.53	0.00	0.83	0.04	S	gi|148544836
Lreu_1736	Protein glcG	2.09	0.00			S	gi|148544942
Lreu_1737	Cobalamin adenosyltransferase family protein	2.17	0.00			S	gi|148544943
Lreu_0240	Protein tyrosine phosphatase (EC 3.1.3.48)			0.85	0.03	T	gi|148543477
Lreu_0677	LexA repressor (EC 3.4.21.88)	1.29	0.00	1.46	0.00	T	gi|148543909
Lreu_0834	Portal protein	1.33	0.01	1.68	0.00	T	gi|148544064
Lreu_1007	Transcription regulator, crp family	-0.82	0.04			T	gi|148544234
Lreu_1731	Protein tyrosine phosphatase (EC 3.1.3.48)	1.88	0.00			T	gi|148544937
Lreu_1115	ATP-dependent endopeptidase clp proteolytic subunit clpP (EC 3.4.21.92)	2.22	0.00	1.66	0.00	U	gi|148544339
Lreu_0812	Phage protein	1.96	0.00	1.65	0.01	V	gi|148544042
Lreu_1121	Phage endonuclease	1.88	0.01	1.37	0.02	V	gi|148544345
Lreu_1138	Phage protein	1.96	0.00	1.65	0.01	V	gi|148544362
Lreu_0178		0.92	0.02	1.38	0.00		gi|148543415
Lreu_0802		1.52	0.00	1.76	0.00		gi|148544032
Lreu_0806		1.84	0.00	1.73	0.00		gi|148544036
Lreu_0813	Phage replication protein	1.97	0.00	1.56	0.00		gi|148544043
Lreu_0814		2.03	0.00	1.57	0.00		gi|148544044
Lreu_0815		1.98	0.00	1.58	0.01		gi|148544045
Lreu_0816		1.92	0.00	1.82	0.00		gi|148544046
Lreu_0821		1.91	0.00	1.73	0.00		gi|148544051
Lreu_0822		1.74	0.00	1.58	0.00		gi|148544052
Lreu_0823		1.73	0.00	1.70	0.00		gi|148544053
Lreu_0827		1.82	0.00	1.95	0.00		gi|148544057
Lreu_0829		1.10	0.01	1.91	0.00		gi|148544059
Lreu_0830		0.99	0.02	1.79	0.00		gi|148544060
Lreu_0832		1.22	0.01	1.80	0.00		gi|148544062

Locus	Function[b]	CDM		CDMglycerol		COG	Accession number
		M[c]	p[d]	M[c]	p[d]		
Lreu_0835	Phage protein	1.37	0.01	1.57	0.00		gi|148544065
Lreu_0836		1.43	0.00	1.44	0.00		gi|148544066
Lreu_0837		1.15	0.03	1.63	0.00		gi|148544067
Lreu_0839	Phage protein	1.26	0.03	1.66	0.01		gi|148544069
Lreu_0840		1.34	0.02	1.79	0.00		gi|148544070
Lreu_0841	Phage protein	1.18	0.04	1.67	0.01		gi|148544071
Lreu_0842		1.29	0.03	1.58	0.01		gi|148544072
Lreu_0843	Phage protein	1.32	0.01	1.64	0.00		gi|148544073
Lreu_0844	Phage protein	1.31	0.01	1.59	0.00		gi|148544074
Lreu_0845		1.51	0.01	1.49	0.01		gi|148544075
Lreu_0847		1.62	0.00	1.57	0.00		gi|148544077
Lreu_0848	Phage protein	1.58	0.00	1.44	0.00		gi|148544078
Lreu_0850		1.49	0.00	1.45	0.00		gi|148544080
Lreu_0851		1.44	0.00	1.55	0.00		gi|148544081
Lreu_0852		1.38	0.04				gi|148544082
Lreu_0854		1.35	0.00	1.56	0.00		gi|148544084
Lreu_0856		1.26	0.01	1.43	0.00		gi|148544086
Lreu_0857				1.17	0.01		gi|148544087
Lreu_1095		1.91	0.01	1.55	0.01		gi|148544319
Lreu_1096		1.96	0.00	1.34	0.00		gi|148544320
Lreu_1097		1.43	0.01	1.47	0.00		gi|148544321
Lreu_1098		1.26	0.01	1.43	0.00		gi|148544322
Lreu_1099		1.35	0.00	1.56	0.00		gi|148544323
Lreu_1100		1.32	0.01	1.43	0.00		gi|148544324
Lreu_1101		1.38	0.04				gi|148544325
Lreu_1102		1.44	0.00	1.55	0.00		gi|148544326
Lreu_1103		1.82	0.00	1.41	0.00		gi|148544327
Lreu_1105		2.11	0.05				gi|148544329
Lreu_1108		2.45	0.00	1.24	0.01		gi|148544332
Lreu_1113	Phage protein	2.20	0.00	1.66	0.00		gi|148544337
Lreu_1122		1.46	0.01	1.12	0.01		gi|148544346
Lreu_1127		1.68	0.00	1.69	0.00		gi|148544351
Lreu_1140		1.57	0.04	1.81	0.02		gi|148544364
Lreu_1143		1.78	0.00	1.73	0.00		gi|148544367
Lreu_1630	Hypothetical protein	1.08	0.01	0.89	0.01		gi|148544837
Lreu_1739		1.79	0.00				gi|148544945
Lreu_1832	Histidine decarboxylase (EC 4.1.1.22)	-1.67	0.03	-1.95	0.01		gi|148545038
Lreu_1838				1.16	0.02		gi|148545044
Lreu_1855				1.12	0.05		gi|148545060

[a] Transcripts were considered for analysis if p-value < 0.05 and M > |0.585|.
[b] Annotation retrieved from ERGO (http://ergo.integratedgenomics.com/ERGO) [16].
[c] M, $\log_2$(intensity of signal of *L. reuteri* pNZ7748/intensity of signal of *L. reuteri* pNZ7021).
[d] p, p-value.

Table S2. Distribution of transcripts listed in Table S1 throughout categories of clusters of orthologous groups (COG)* [35].

COG category		up-regulated when *lreu_1750* is overexpressed (%)	down-regulated when *lreu_1750* is overexpressed (%)	regulated *lreu_1750* is overexpressed (%)
C – Energy production and conversion (59)	CDM	11.86	0.00	11.86
	CDMglycerol	1.69	6.78	8.47
D – Cell cycle control, cell division, chromosome partitioning (17)	CDM	0.00	0.00	0.00
	CDMglycerol	0.00	0.00	0.00
E – Amino acid transport and metabolism (130)	CDM	2.31	0.00	2.31
	CDMglycerol	0.77	1.54	2.31
F – Nucleotide transport and metabolism (75)	CDM	0.00	0.00	0.00
	CDMglycerol	0.00	2.67	2.67
G – Carbohydrate transport and metabolism (80)	CDM	3.75	0.00	3.75
	CDMglycerol	0.00	10.00	10.00
H – Coenzyme transport and metabolism (71)	CDM	9.86	0.00	9.86
	CDMglycerol	7.04	1.41	8.45
I – Lipid transport and metabolism (34)	CDM	2.94	0.00	2.94
	CDMglycerol	0.00	0.00	0.00
J – Translation, ribosomal structure and biogenesis (139)	CDM	0.00	0.00	0.00
	CDMglycerol	0.00	0.00	0.00
K – Transcription (93)	CDM	6.45	0.00	6.45
	CDMglycerol	7.53	0.00	7.53
L – Replication, recombination and repair (176)	CDM	3.41	0.00	3.41
	CDMglycerol	2.27	0.00	2.27
M – Cell wall/membrane/envelope biogenesis (80)	CDM	1.25	0.00	1.25
	CDMglycerol	1.25	0.00	1.25
N – Cell motility (1)	CDM	0.00	0.00	0.00
	CDMglycerol	0.00	0.00	0.00
O – Posttranslational modification, protein turnover, chaperones (42)	CDM	0.00	0.00	0.00
	CDMglycerol	2.38	2.38	4.76
P – Inorganic ion transport and metabolism (58)	CDM	3.45	0.00	3.45
	CDMglycerol	8.62	3.45	12.07
Q – Secondary metabolites biosynthesis, transport and catabolism (26)	CDM	34.62	0.00	34.62
	CDMglycerol	3.85	0.00	3.85
R – General function prediction only (235)	CDM	1.70	0.00	1.70
	CDMglycerol	0.85	0.43	1.28
S – Function unknown (149)	CDM	8.72	0.00	8.72
	CDMglycerol	7.38	0.67	8.05
T – Signal transduction mechanisms (53)	CDM	5.66	1.89	7.55
	CDMglycerol	5.66	0.00	5.66
U – Intracellular trafficking, secretion, and vesicular transport (23)	CDM	4.35	0.00	4.35
	CDMglycerol	4.35	0.00	4.35
V – Defense mechanisms (34)	CDM	8.82	0.00	8.82
	CDMglycerol	8.82	0.00	8.82

* Values refer to percentage of transcripts from each category that is differentially expressed.

Table S3. Complete list of loci from *L. reuteri* ATCC PTA 6475 that are differentially expressed by the disruption of the putative PocR [a].

Locus	Function[b]	M[c]	*p*[d]
**NT01LR0069**	**PduH protein**	-5.86	0.00
**NT01LR0064**	**propanediol utilization protein PduB**	-5.84	0.00
**NT01LR0072**	**propanediol utilization protein**	-5.83	0.00
**NT01LR0068**	**glycerol dehydratase reactivation factor largesubunit**	-5.83	0.00
NT01LR1323	Ham1 family	-5.69	0.00
**NT01LR0065**	**glycerol dehydratase large subunit**	-5.51	0.00
**NT01LR0067**	**propanediol dehydratase small subunit**	-5.42	0.00
**NT01LR0070**	**propanediol utilization protein**	-5.38	0.00
**NT01LR0066**	**propanediol dehydratase medium subunit**	-5.28	0.00
**NT01LR0073**	**propanediol utilization protein**	-5.20	0.00
**NT01LR0063**	**propanediol utilization protein PduA**	-5.06	0.00
NT01LR0075	*ATP:cob(I)alamin adenosyltransferase*	-4.99	0.00
**NT01LR0071**	**propanediol utilization protein PduA**	-4.97	0.00
**NT01LR0080**	**propanediol utilization protein**	-4.83	0.00
**NT01LR0077**	**ethanolamine utilization protein EutE**	-4.65	0.00
**NT01LR0076**	**propanediol utilization protein**	-4.54	0.00
**NT01LR0081**	**protein tyrosine/serine phosphatase**	-4.51	0.00
ackA_1	acetate kinase	-4.39	0.00
**NT01LR0078**	**alcohol dehydrogenase class IV**	-4.34	0.00
**NT01LR0074**	**propanediol utilization protein**	-4.02	0.00
**NT01LR0060**	**glycerol uptake facilitator protein**	-3.74	0.00
cbiD	*cobalamin biosynthesis protein CbiD*	-3.54	0.00
NT01LR1345	conserved hypothetical protein	-3.51	0.03
folB	dihydroneopterin aldolase	-3.27	0.02
NT01LR1358	*cobalt ABC transporter*	-3.21	0.01
rimM	16S rRNA processing protein RimM	-3.18	0.03
NT01LR1363	*sirohydrochlorin cobaltochelatase*	-3.06	0.00
NT01LR1249	ribosomal large subunit pseudouridine synthase,RluD subfamily	-3.01	0.02
NT01LR1173	D-serine/D-alanine/glycine transporter	-2.95	0.03
NT01LR0418	polysaccharide biosynthesis protein, putative	-2.81	0.03
NT01LR1346	conserved hypothetical protein	-2.79	0.04
NT01LR1360	*ABC-type cobalt transport system*	-2.67	0.00
NT01LR1715	integral membrane protein	-2.56	0.05
nhaC_2	Na+/H+ antiporter NhaC	-2.41	0.05
NT01LR0084	integral membrane protein	-2.37	0.00
NT01LR1369	*precorrin-8w decarboxylase*	-2.22	0.05
cbiQ	*cobalt ABC transporter, permease protein CbiQ*	-2.21	0.04
NT01LR1609	transcriptional regulator, MarR family	-2.19	0.05
cobI	*precorrin-2 C20-methyltransferase*	-2.18	0.01
NT01LR1598	enoyl-[acyl-carrier-protein] reductase [NADH]	-2.13	0.05
NT01LR1326	conserved hypothetical protein	-1.88	0.04
NT01LR1066	extracellular surface protein	-1.87	0.05
rpmB	ribosomal protein L28	-1.72	0.01
rpsJ	ribosomal protein S10	-1.71	0.02
NT01LR1438	ribonucleoside-diphosphate reductase [multifunctional]	-1.70	0.03
mfd	transcription-repair coupling factor	-1.68	0.05
NT01LR0719	1,3-propanediol dehydrogenase	-1.61	0.01
NT01LR1625	extracellular surface protein	-1.51	0.05
rpsG	ribosomal protein S7	-1.47	0.01
rplC	ribosomal protein L3	-1.42	0.02
rpsL	ribosomal protein S12	-1.38	0.02
rplB	ribosomal protein L2	-1.36	0.03
NT01LR1564	50S ribosomal protein L5	-1.33	0.01
nusG	transcription termination/antitermination factorNusG	-1.31	0.05
cobM	*precorrin-4 C11-methyltransferase*	-1.28	0.05
cbiM	*cobalamin biosynthesis protein CbiM*	-1.28	0.04
rpsF	ribosomal protein S6	-1.27	0.03
rplW	ribosomal protein L23	-1.24	0.02
hemC	*porphobilinogen deaminase*	-1.20	0.03
rplO	ribosomal protein L15	-1.18	0.03

Locus	Function[b]	M[c]	p[d]
rpsS	ribosomal protein S19	-1.17	0.02
NT01LR0917	acetyltransferase, gnat family	-1.15	0.04
rplD	ribosomal protein L4/L1 family	-1.15	0.05
**NT01LR0061**	**ethanolamine utilization protein**	-1.14	0.02
NT01LR0341	cell division initiation protein	-1.13	0.03
NT01LR0775	ribosomal protein	-1.08	0.05
rpsT	ribosomal protein S20	-1.07	0.03
rplX	ribosomal protein L24	-1.07	0.02
rpsQ	ribosomal protein S17	-1.07	0.02
rpmD	ribosomal protein L30	-1.06	0.03
NT01LR0108	single-stranded DNA-binding protein	-1.04	0.04
rplN	ribosomal protein L14	-1.04	0.03
NT01LR1582	type IV pilus prepilin peptidase PilD	-1.03	0.03
NT01LR1280	membrane protein	-1.03	0.04
NT01LR0510	ribonucleic acid-binding domain protein	-0.99	0.04
NT01LR0218	metallo-beta-lactamase superfamily hydrolase	-0.98	0.05
NT01LR1117	bifunctional autolysin	-0.97	0.05
rplR	ribosomal protein L18	-0.96	0.05
gnd	6-phosphogluconate dehydrogenase,decarboxylating	-0.96	0.03
NT01LR1561	50S ribosomal protein L6	-0.94	0.03
rpmC	ribosomal protein L29	-0.94	0.05
rpmF	ribosomal protein L32	-0.88	0.04
NT01LR1099	UDP-N-acetylmuramyl tripeptide synthase	-0.86	0.05
NT01LR0802	alkaline shock protein	-0.84	0.05
NT01LR0402	lr0524	-0.84	0.05
rseP	RIP metalloprotease RseP	-0.83	0.05
NT01LR0050	ribonucleotide reduction protein	-0.83	0.05
rpsN	ribosomal protein S14p/S29e	-0.80	0.05
NT01LR0082	phosphoglycerate mutase	-0.79	0.05
NT01LR1486	conserved hypothetical protein	0.86	0.04
NT01LR1412	acetyltransferase, gnat family	0.87	0.05
NT01LR0639	cysteine-rich domain of 2-hydroxy-acid oxidaseGlcF	0.87	0.04
NT01LR0452	response regulator of the LytR	0.88	0.05
NT01LR1683	hypothetical protein	0.92	0.04
NT01LR1487	protein lp_2275	0.92	0.05
NT01LR0640	iron-sulfur cluster binding protein	0.95	0.04
NT01LR1802	molybdopterin biosynthesis protein, D chain	0.98	0.04
NT01LR1685	conserved hypothetical protein	1.00	0.04
NT01LR0139	YbaK	1.01	0.02
NT01LR1679	hypothetical protein	1.03	0.03
NT01LR1410	aminopeptidase C	1.04	0.05
NT01LR0641	YkgG family protein	1.07	0.05
NT01LR1677	prophage Lp1 protein 19	1.08	0.04
NT01LR1672	hypothetical protein	1.08	0.03
NT01LR1681	hypothetical protein	1.11	0.03
NT01LR1688	conserved hypothetical protein	1.14	0.01
NT01LR1659	cytosine deaminase	1.15	0.05
NT01LR1686	hypothetical protein	1.17	0.01
NT01LR0150	DNA translocase FtsK	1.18	0.04
NT01LR1979	conserved domain	1.18	0.05
NT01LR1675	hypothetical protein	1.19	0.02
NT01LR1485	conserved protein YrzB	1.22	0.04
NT01LR0760	3-dehydroquinate synthase	1.22	0.01
NT01LR1680	hypothetical protein	1.23	0.01
NT01LR1674	transcriptional regulator	1.25	0.01
NT01LR1671	hypothetical protein	1.26	0.02
NT01LR1107	lipoprotein, putative	1.31	0.05
NT01LR0613	bacilysin biosynthesis oxidoreductase BacC	1.32	0.04
NT01LR0973	conserved domain protein	1.32	0.02
cytX	probable hydroxymethylpyrimidine transporterCytX	1.35	0.02
NT01LR0884	HypQ3	1.39	0.02
NT01LR0268	Low molecular weightprotein-tyrosine-phosphatase YfkJ	1.42	0.02
narH	nitrate reductase, beta subunit	1.42	0.03

Locus	Function[b]	M[c]	p[d]
NT01LR0758	conserved hypothetical protein	1.44	0.01
narJ	nitrate reductase molybdenum cofactor assemblychaperone	1.47	0.03
NT01LR1678	DnaA analog	1.49	0.03
NT01LR0270	putative Probable protease HtpX	1.50	0.03
NT01LR0960	alkaline shock protein	1.60	0.01
NT01LR1380	conserved hypothetical protein	1.64	0.03
mscL	large conductance mechanosensitive channelprotein	1.73	0.00
NT01LR1713	conserved hypothetical protein	1.79	0.00
NT01LR0849	hypothetical protein	1.82	0.04
NT01LR1031	DNA protection during starvation protein	2.02	0.00
NT01LR0962	conserved hypothetical protein	2.06	0.00
NT01LR1423	aminotransferase, class I and II family protein	2.18	0.03
NT01LR0732	histone acetyltransferase	2.34	0.03
NT01LR1872	bifunctional thioredoxin reductase/thioredoxin	2.49	0.00
rnd2		4.58	0.04
NT01LR0062	transcriptional regulator	5.24	0.00

[a] Transcripts were considered for analysis if *p*-value ≤ 0.05 and M > |0.585|. Loci located within the genetic island are formatted in **bold** if they belong to the pdu operon and in *italic* if they encode vitamin B_{12} synthesis enzymes.

[b] Annotation retrieved from GenBank.

[c] M, $\log_2$(intensity of signal of *L. reuteri* 6475::pORIpocR/intensity of signal of *L. reuteri* 6475).

[d] *p*, *p*-value.
